# Comparison of efficacy of continuous epidural block and pulsed radiofrequency to the dorsal root ganglion for management of pain persisting beyond the acute phase of herpes zoster

**DOI:** 10.1371/journal.pone.0183559

**Published:** 2017-08-21

**Authors:** Eung Don Kim, Young In Lee, Hue Jung Park

**Affiliations:** 1 Department of Anesthesiology and Pain Medicine, School of Medicine, The Catholic University of Korea, Daejeon St. Mary’s Hospital, Daejeon, Korea; 2 Department of Anesthesiology and Pain Medicine, School of Medicine, The Catholic University of Korea, Seoul St. Mary’s Hospital, Seoul, Korea; University of Pittsburgh School of Medicine, UNITED STATES

## Abstract

**Background:**

There is little evidence regarding the effectiveness of intervention methods in the treatment of zoster-related pain (ZAP) after the acute phase of zoster. Generally, if ZAP remains after more than 180 days from its onset, the likelihood of pain reduction is very low; this condition is considered as a “well established” post-herpetic neuralgia (PHN). Although the clinical efficacy of intrathecal steroid injection and spinal cord stimulation (SCS) for ZAP management has been reported, these interventions are not widely used due to inherent disadvantages. Continuous epidural block is widely used in clinical practice, and the effectiveness of pulsed radiofrequency (PRF) to the dorsal root ganglion (DRG) in the treatment of ZAP already has been reported.

**Objectives:**

The purpose of this study was to compare the clinical efficacy of continuous epidural block and DRG PRF beyond acute phase of zoster, bur before PHN was well established (from 30 days to180 days after zoster onset).

**Study design:**

Retrospective comparative study.

**Methods:**

A total of 42 medical records were analyzed. Patients were divided into two groups according to the type of procedure utilized: continuous epidural block (continuous epidural group) and DRG PRF (PRF group). The clinical efficacy of the procedure was evaluated using a numeric rating scale (NRS) and the medication dose before and 1 to 6 months after the procedure.

**Results:**

There was a significant decrease in the NRS value with time in both groups. However, this decrease was more significant in the PRF group than in the continuous epidural group. The medication doses decreased significantly in the PRF group over time, but not in the continuous epidural group. The rate of clinically meaningful PHN (NRS≥3) was also lower in the PRF group than in the continuous epidural group.

**Conclusions:**

This study revealed that DRG PRF was more effective than a continuous epidural block in treating ZAP after the acute phase of zoster. A neuromodulation method such as DRG PRF may be a useful option for reducing the progression of neuropathic changes caused by the persistent transmission of a pain signal after the acute phase of zoster.

## Introduction

Herpes zoster is an infectious disease caused by reactivation of latent varicella zoster virus (VZV), with a lifetime incidence of 30% [1]. Post-herpetic neuralgia (PHN) is the most common complication of herpes zoster, and can impair quality of life due to pain [2].

The acute phase of herpes zoster is usually defined within 30 days after onset of the rash, and various nerve blocks including epidural block are used in the acute stage to prevent PHN [3–5]; favorable outcomes for epidural or paravertebral block have been reported [6–9]. If adequate pain reduction is not achieved within the acute phase of herpes zoster, a neuropathic process, such as central sensitization due to persistent nociceptive signaling from damaged neurons, may be initiated. This is one of the major causes of PHN.

The presence of zoster-associated pain (ZAP) in spite of various nerve blocks during the acute phase of herpes zoster can be a challenge to pain physicians. In addition, few interventions for managing pain in the PHN period are supported by evidence [10,11].

The exact discriminative time point for PHN has not yet been standardized. Various criteria have been used, from 30 days to 180 days after zoster onset. If pain persists for more than 180 days after zoster onset, the likelihood of pain reduction is very low and such a condition is considered "well established" PHN [3–5]. Therefore, it is advisable to actively attempt various treatment modalities for pain control before the condition progresses to a recalcitrant state.

Intrathecal steroid injection or spinal cord stimulation (SCS) has been reported to be effective for ZAP control. However, there are also concerns about the risk of potential complications and high medical costs associated with each procedure, respectively. Therefore, it would be beneficial to find an intervention method that can be easily and safely applied in clinical practice.

Continuous epidural block is often used in clinical practice in cases where pain is refractory to conservative treatment. The usefulness of repetitive epidural block using an indwelling catheter in acute herpes zoster has been reported [6], as well as the long-term effects of continuous nerve block with local anesthetic infusion for chronic neuropathic conditions such as complex regional pain syndrome (CRPS) or PHN [12,13]. Repetitive infusion of local anesthetics into the paravertebral space in PHN has also been reported to be effective [14]. Although there was lack of objective evidence, continuous epidural block has occasionally been used to relieve pain that persisted after the acute phase of zoster [5].

Pulsed radiofrequency (PRF) is a variant of thermal radiofrequency that applies pulsed current to limit heat generation to less than 42°C [15,16], creating little risk of thermal or nerve injury [17,18]. Recently, the use of PRF has increased in many chronic pain conditions, including trigeminal neuralgia, chronic spinal pain, and musculoskeletal pain [19–21].

PRF also has been reported to be useful in controlling pain during PHN and herpes zoster [22–24]. In particular, the fact that latent VZV initiates reactivation in the dorsal root ganglion (DRG) suggests that the DRG is an appropriate target for ZAP management. A few studies have reported the usefulness of DRG PRF in ZAP [23,24]. However, these studies were observational in nature without comparative groups such as other interventions.

Although continuous epidural block is often used for ZAP management when pain persists after the acute phase of zoster in clinical practice, and DRG PRF was also reported to be useful for ZAP control in PHN or subacute herpes zoster, the clinical effects of these procedures in ZAP management after the acute phase of zoster have not been compared.

We retrospectively compared the efficacy of continuous epidural block and DRG PRF for ZAP management beyond the acute phase of zoster, but before PHN was well established (between 30 and 180 days after zoster onset).

## Methods

### Participants

We analyzed the medical records of patients who underwent continuous epidural block or DRG PRF to manage ZAP from January 2010 to September 2016. In this analysis, only the medical records of patients who underwent the procedure between 30 and 180 days after zoster onset were included. We classified patients with continuous epidural block as the continuous epidural group and those with DRG PRF as the PRF group.

Trigeminal-nerve-involved zoster or follow-up loss within 6 months after the procedure was excluded from the analysis. We also excluded those who did not receive appropriate antiviral treatment during the acute phase of herpes zoster. To accurately compare the effects of the two procedures, we excluded cases where both procedures were performed between 30 and 180 days of zoster onset.

This retrospective analysis was approved by the Institutional Ethics Committee of Daejeon St. Mary’s Hospital, Republic of Korea (DC17RESI0027).

### Procedure

#### Continuous epidural block

After placing the patient in the prone position, an 18-gauge Tuohy needle was introduced into the interlaminar space at the second or third level below the target level under fluoroscopic guidance.

**Fig 1:**
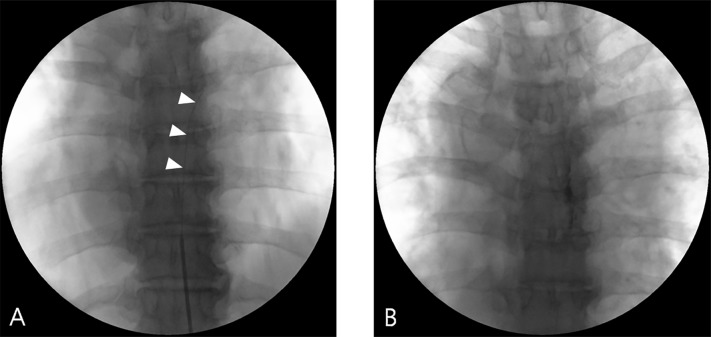
Fluoroscopic images of continuous epidural block. Insertion of an epidural catheter via Tuohy needle (A) and confirmation of catheter tip position using contrast media (B). White arrowheads indicate the catheter inserted through a Tuohy needle.

The epidural space was confirmed using the loss of resistance (LOR) technique; then a 20- gauge epidural catheter was inserted through the Tuohy needle and placed at the target level (Fig 1).

After confirming the position of the catheter tip, 0.187% ropivacaine was infused at a rate of 1 ml per hour. Ropivacaine concentration and rate of administration were adjusted by the degree of pain relief or side effects. When appropriate pain relief was achieved, the catheter was removed. The period of catheterization was limited to within 2 weeks, due to concerns regarding infection.

#### DRG PRF

The patient was placed in the prone position and the fluoroscopy was slightly rotated to the lesion side. A 22-gauge, 10-cm electrode with a 10-mm active tip (Radionics Inc., Burlington, MA, USA) was placed near the target DRG under fluoroscopic guidance.

**Fig 2:**
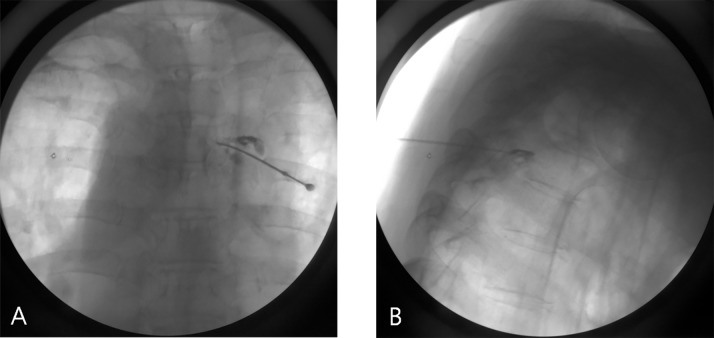
Fluoroscopic images of DRG PRF. Fluoroscopic anteroposterior view (A) and lateral view (B) of the DRG PRF.

The needle tip was placed under the pedicle in the anteroposterior view and in the posterocranial portion of the intervertebral foramen in the lateral view for fluoroscopic imaging (Fig 2).

Sensory stimulation was performed using a 50-Hz current. If a tingling sensation was observed in the affected dermatome below 0.5 V, the position of the needle was considered appropriate.

After confirming the needle position, PRF of 42ºC (20 milliseconds, 2 Hz, 45 V) was applied for 360 seconds. Impedance was maintained at less than 500 Ω throughout the procedure.

### Data collection

The following data were collected and analyzed: sex, age, involved dermatome, days from zoster onset to the procedure, types of procedures performed during acute herpes zoster, numerical rating scale (NRS) before the procedure, NRS 1 to 6 months after the procedure, and doses of anticonvulsants and analgesics before and 1 to 6 months after the procedure.

We also investigated whether an additional nerve block was performed during the 6-month follow-up period after each procedure.

### Outcome measures

The analgesic effect of each procedure was assessed by the NRS, as well as the dose of anticonvulsants and analgesics. The NRS and dose of anticonvulsants and analgesics were compared between groups both before the procedure, and 1 to 6 months after the procedure.

For ease of analysis, anticonvulsant doses were converted to pregabalin-equivalent doses [25,26] and analgesic doses were converted to oral morphine-equivalent doses [27].

In various studies, clinically meaningful PHN was defined as persistent pain with an intensity of three points or more on the NRS [28–30]. In this study, the proportion of clinically meaningful PHN was compared between the two groups 1, 3 and 6 months after the procedure based on the same criterion.

### Statistical analysis

Data normality was evaluated using the Kolmogorov-Smirnov test. The Mann-Whitney U test or the independent t-test was used to compare the outcomes between the groups for continuous variables. Data are presented as mean ± standard deviation (SD) for continuous variables. For categorical variables, the Chi-square test or Fisher’s exact test was used.

Repeated measures analysis of variance was used to assess of the alterations in pain medication doses after the procedure over time. All data were analyzed using SPSS version 18.0 (SPSS Inc., Chicago, IL), and *p* values of <0.05 were considered statistically significant.

## Results

**Fig 3:**
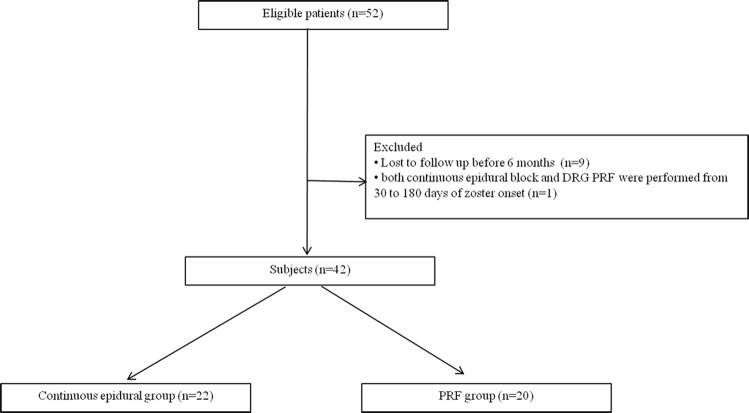
Flow diagram of the study subjects. PRF: pulsed radiofrequency. DRG: dorsal root ganglion.

A total of 52 patient medical records were reviewed; of these, 9 patients were lost to follow-up before 6 months following the procedure. One patient underwent both continuous epidural block and DRG PRF between 30 and 180 days of zoster onset; the medical record of this patient was excluded from the analysis. In summary, the medical records of 42 patients were used for the analysis. Among them, 22 patients underwent continuous epidural block (continuous epidural group) and another 20 patients underwent DRG PRF (PRF group) (Fig 3).

Age, sex, level of involved dermatome, and presence of underlying disease were not significantly different between the two groups. There were also no significant differences between the groups in pre-procedure NRS, dose of medication before the procedure, and types of intervention during the acute herpes zoster phase (Table 1).

**Table 1:** Patient demographic data.

	Continuous epidural group (n = 22)	PRF group (n = 20)	*p*-value
Age, years, mean ± SD	70.41 ± 10.25	68.10 ± 7.99	0.424
Sex, n, male/female	6/16	11/9	0.115
Pre-NRS, mean ± SD	6.73 ± 0.88	6.30 ± 0.98	0.219
Days from zoster onset, mean ± SD	74.09 ± 44.50	68.20 ± 40.53	0.657
Involved dermatome, n			
Cervical, n (%)	2 (9.1%)	2 (10.0%)	0.627
Thoracic, n(%)	19 (86.4%)	18 (90.0%)	
Lumbosacral, n(%)	1 (4.5%)	0 (0%)	
Underlying disease			
Hypertension (HTN), n (%)	6 (27.3%)	8 (40.0%)	0.203
Diabetes mellitus (DM), n (%)	4 (18.1%)	1 (5.0%)	
HTN & DM, n (%)	6 (27.3%)	2 (10.0%)	
None, n (%)	6 (27.3%)	9 (45.0%)	
Procedures during the acute stage of herpes zoster			
Continuous epidural catheterization, n (%)	5 (22.7%)	11(55.0%)	0.055
Transforaminal epidural block, n (%)	17 (77.3%)	19 (86.4%)	0.187
Doses of medication before the procedure			
Anticonvulsant, pregabaline-equivalent dose, mg	235.23 ± 141.35	228.75 ± 138.67	0.882
Analgesics, oral morphine equivalent dose, mg	30.82 ± 20.47	35.27 ± 21.42	0.496

PRF: pulsed radiofrequency

The mean duration of catheter implantation in the continuous epidural group was 11.182±3.142 days, and the mean concentration of ropivacaine and infusion rates used were 0.224±0.07% and 1.818±0.646 ml/hr, respectively.

**Fig 4:**
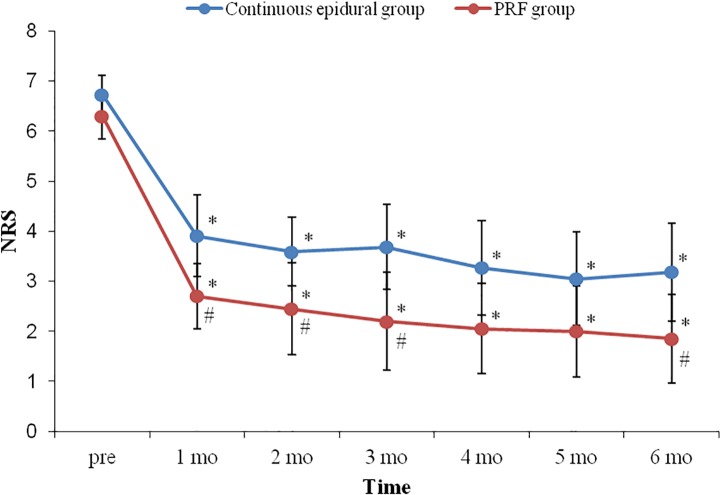
Changes in NRS after continuous epidural block and DRG PRF. Although significant NRS changes throughout the time course were shown in both groups compared to the pre-procedure time point, the reduction in NRS was more profound in the PRF group (p = 0.029). NRS was significantly lower in the PRF group one to three months and six months after the procedure than in the continuous epidural group. Blue line with circle box and red line with circle box indicate NRS changes in continuous epidural group andPRF group respectively. NRS: numerical rating scale. DRG: dorsal root ganglion. PRF: pulsed radiofrequency. *: p<0.05 compared to pre-procedure NRS. #: p<0.05 compared to the continuous epidural group.

Although significant NRS changes through the time course were shown in both groups compared to the time point of pre-procedure, the reduction in NRS was more profound in the PRF group (*p* = 0.029). NRS values were significantly lower in the PRF group from 1 month to 3 months and 6 months after the procedure than those in the continuous epidural group (Fig 4).

**Fig 5:**
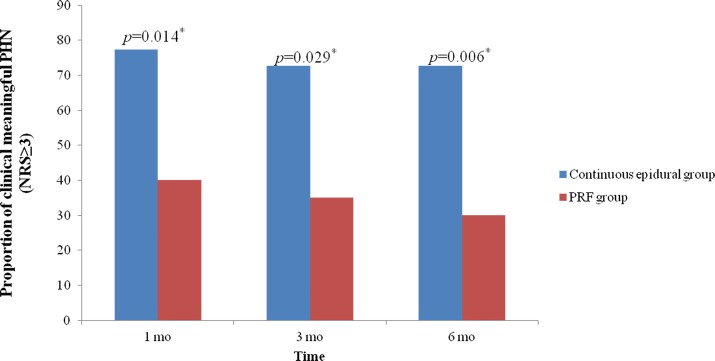
Rate of clinically meaningful PHN (NRS ≥3). NRS: numerical rating scale. PRF: pulsed radiofrequency. *: p<0.05.

The proportion of clinically meaningful PHN (NRS ≥3) was significantly higher in the continuous epidural group at intervals of 1, 3 and 6 months (Fig 5).

**Fig 6:**
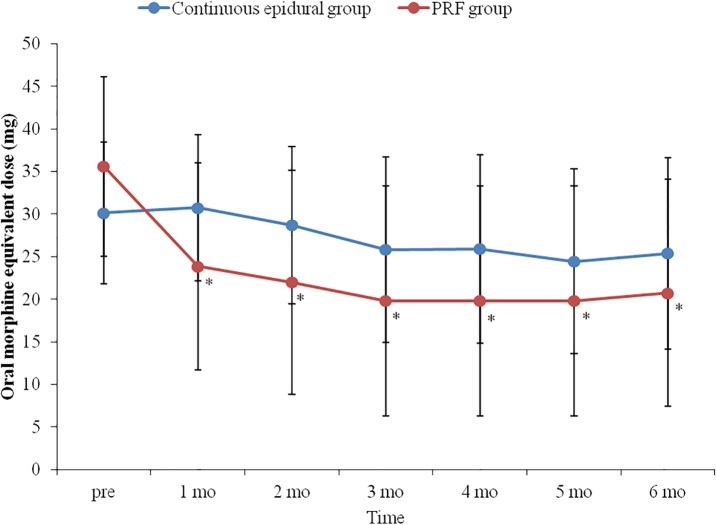
Changes in doses of analgesics with time. In the PRF group, doses of analgesics one to six months after DRG PRF were significantly decreased compared to pre-procedure. There were no significant changes in analgesic doses with time in the continuous epidural group. Blue line with circle box and red line with circle box indicate analgesics dose changes in continuous epidural group and PRF group respectively. DRG: dorsal root ganglion. PRF: pulsed radiofrequency. *: p<0.05 compared to pre-procedure.

There was no significant difference in analgesic dose between the two groups. However, in the PRF group, there was a significant decrease in dose in the first month after the procedure compared to the pre-procedure dose, while there were no significant changes at all time points compared to pre-procedure in the continuous epidural group (Fig 6).

**Fig 7:**
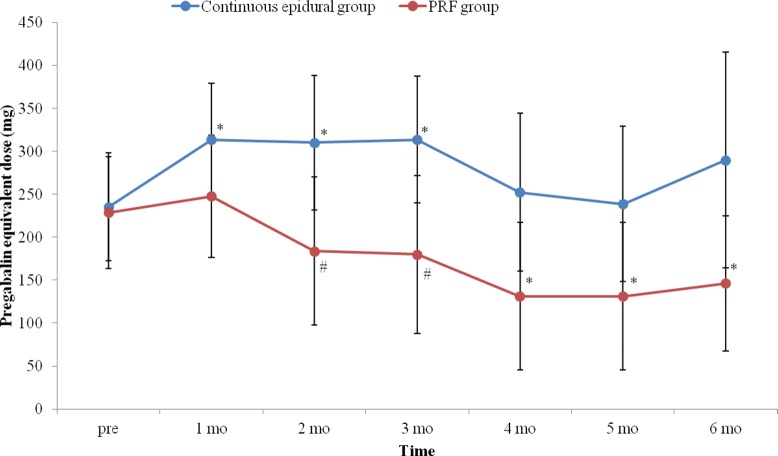
Changes in doses of anticonvulsant with time. There was significantly difference in the anticonvulsant dose changes over time between the two groups (*p* = 0.042). In the continuous epidural group, anticonvulsant doses one to three months after continuous epidural block were significantly increased compared to pre-procedure. However, in the PRF group, anticonvulsant doses were significantly decreased four months post-procedure compared to pre-procedure. Blue line with circle box and red line with circle box indicate anticonvulsant dose changes in continuous epidural group and PRF group respectively. DRG: dorsal root ganglion. PRF: pulsed radiofrequency. *: p<0.05 compared to pre-procedure. #: p<0.05 compared to the continuous epidural group.

The anticonvulsant doses were significantly higher in the continuous epidural group 2 months and 3 months after the procedure. The changes in anticonvulsant dose over time were significantly different between the two groups (*p* = 0.042). In the PRF group, there was a significant decrease in anticonvulsant dose at intervals of 4, 5 and 6 months compared to the pre-procedure dose, while in the continuous epidural group, it was significantly increased until 3 months after the procedure (Fig 7).

The proportion of patients requiring additional nerve blocks for ZAP control after continuous epidural block or DRG PRF was lower in the PRF group than in the continuous epidural group; however, this difference was not statistically significant (continuous epidural group: 11/22, 50.0% vs. DRG PRF group: 5/20, 25.0%; *p* = 0.096).

There were no serious complications associated with the procedure in either group. However, 36.3% (8/22) of patients in the continuous epidural group showed evidence of mild complications,including catheter insertion site pain, headache, dizziness, dysuria, constipation, and motor weakness. In the case of one patient, the continuous epidural block was discontinued due to severe pain at the catheter insertion site. In contrast, in the PRF group, only one patient complained of pain at the procedure site, and it improved within a few days. This difference in the incidence of complications between the groups was significant (*p* = 0.013).

## Discussion

In the present study, pain intensity decreased with time in both groups. However, pain reduction was moreprofound in the PRF group compared to the continuous epidural group. The proportion of patients with clinically meaningful PHN (NRS ≥3) was also lower in the PRF group.

Significant shortening of ZAP duration in continuous epidural block in acute herpes zoster has been reported [31]. Even after the acute phase has elapsed, before PHN is well established, blocking the delivery of nociceptive signals to the central nervous system including the dorsal horn by continuous infusion of local anesthetics may prevent further progression of neuropathic changes. Therefore, a continuous epidural block extending beyond the acute phase of zoster may be an option for symptom palliation and prevention of further progression to PHN.

This study was the first to analyze the results of a continuous epidural block applied after the acute period of herpes zoster; however, the clinical results of the continuous epidural group were unsatisfactory compared to the PRF group.

We started infusion with a low concentration of 0.187% ropivacaine, which is a relatively low concentration of local anesthetic compared to that utilized in other studies. This low concentration of local anesthetic may be the origin of the inferior outcomes of continuous epidural block in this analysis. However, it has been reported that the concentration of local anesthetic required to inhibit ectopic discharge from a damaged nerve is lower than the concentration required to block normal nerve conduction [32, 33].

Moreover, the most common zoster-affected levels are thoracic dermatomes, and patients who undergo continuous epidural block to control ZAP are usually elderly. In these patients, persistent high rates of infusion and high concentrations of local anesthetic administered over several days may lead to extensive hemodynamic changes, and these changes can be particularly dangerous in the elderly. Therefore this can be a barrier to increasing the concentration and infusion rate of local anesthetics.

On the other hand, DRG PRF may have a relatively lower risk of hemodynamic changes than continuous epidural block. Although the exact mechanism of the analgesic effect of PRF has not been fully elucidated, recent studies have reported increases in c-fos expression [34, 35], evidence of selective lesioning of small A, δ, and C fibers [36], and increased synaptic changes in transmission [37]. These results suggest that PRF is a kind of neuromodulation modality [38]. This modification of neural tissue may contribute to the long-term effects of PRF.

In the present study, the decrease in the NRS value was greater in the PRF group than in the continuous epidural group, and doses of anticonvulsants and analgesics tended to decrease with time in the PRF group, while this tendency was not found in the continuous epidural group. These results may support the long-term effects of PRF.

Spinal cord stimulation (SCS) is also a representative method of neuromodulation. The usefulness of SCS in the treatment of subacute herpes zoster or PHN has been reported [39]. However, although the thoracic level is the most common zoster-affected dermatome, appropriate stimulation can be difficult because the depth of the cerebrospinal fluid is greatest at the thoracic level. Moreover, medical costs are high for SCS.

Although intrathecal steroid injection has been reported to be effective during the PHN period [40], the clinical efficacy of intrathecal steroid injections has been questioned, consideringprevious animal studies and the potential risks of this procedure such as adhesive arachnoiditis [41,42].

Because of concerns associated with SCS and intrathecal steroid injection, these procedures are unlikely to become standard treatments for PHN or subacute herpes zoster. Continuous epidural block also poses the risk of infection due to the presence of an indwelling catheter. Moreover, epidural catheterization generally requires hospitalization.

In contrast, DRG PRF is less likely to cause a fatal infection than the above-mentioned procedures, and can easily be performed in an outpatient clinic setting without hospitalization. In the present analysis, the incidence of mild complications was significantly lower in the PRF group than in the continuous epidural group.

This was a retrospective study based on the analysis of medical records, and medication data relied on prescription records. In many cases, patients reported self-tapering of medication due to reduced pain. Therefore, there may be a difference between the actual dose taken and the prescribed medication dose, which, together with the small sample size, may be a limitation of the present study.Additionally, since this study was a retrospective analysis, we could not standardize the medications. Because of this, it is possible that there was a statistical error in the comparison of medication doses between groups.The absence of a "no-procedural" control group can also weaken the conclusions of the presentanalysis, which is another limitation of our study.

## Conclusions

In conclusion, the PRF group showed a significant decrease in ZAP over time compared with the continuous epidural block group, and the rate of clinically meaningful PHN in the PRF group was significantly lower than that in the continuous epidural block group. This is the first study to compare the clinical outcomes of different intervention methods applied in the period following the acute phase of herpes zoster. Based on these results, in cases with progressive neuropathic changes such as those following the acute stage of herpes zoster,a neuromodulation modality such as DRG PRF may be more effective for pain management than continuous blockade of nociceptive input.

Further well-controlled, prospective trials with appropriate sample sizes are needed to confirm these findings.

## Supporting information

S1 File File containing raw data of study subjects. (XLSX)
